# Development and validation of anthropometric equations to estimate body composition in adult women

**DOI:** 10.25100/cm.v49i2.3643

**Published:** 2018-06-30

**Authors:** Juan C. Aristizabal, Alejandro Estrada-Restrepo, Argenis Giraldo García

**Affiliations:** 1 Physiology and Biochemistry Research Group-PHYSIS, Universidad de Antioquia, Medellin, Colombia.; 2 School of Nutrition and Dietetics, Universidad de Antioquia, Medellin, Colombia.; 3 Demography and Health Research Group, Universidad de Antioquia, Medellin, Colombia.

**Keywords:** Women, body composition, obesity, anthropometry, skinfold thickness, body weight, Mujeres, composición corporal, obesidad, antropometría, pliegues cutáneos, peso corporal

## Abstract

**Objective:**

To develop anthropometric equations to predict body fat percentage (BF%).

**Methods:**

In 151 women (aged 18-59) body weight, height, eight- skinfold thickness (STs), six- circumferences (CIs), and BF% by hydrodensitometry were measured. Subjects data were randomly divided in two groups, equation-building group (n= 106) and validation group (n= 45). The equation-building group was used to run linear regression models using anthropometric measurements as predictors to find the best prediction equations of the BF%. The validation group was used to compare the performance of the new equations with those of Durnin-Womersley, Jackson-Pollock and Ramirez-Torun.

**Results:**

There were two preferred equations: Equation 1= 11.76 + (0.324 x tricipital ST) + (0.133 x calf ST) + (0.347 x abdomen CI) + (0.068 x age) - (0.135 x height) and Equation 2= 11.37 + (0.404 x tricipital ST) + (0.153 x axilar ST) + (0.264 x abdomen CI) + (0.069 x age) - (0.099 x height). There were no significant differences in BF% obtained by hydrodensitometry (31.5 ±5.3) and Equation 1 (31.0 ±4.0) and Equation 2 (31.2 ±4.0). The BF% estimated by Durning-Womersley (35.8 ±4.0), Jackson-Pollock (26.5 ±5.4) and Ramirez-Torun (32.6 ±4.8) differed from hydrodensitometry (*p* <0.05). The interclass correlation coefficient (ICC) was high between hydrodensitometry and Equation 1 (ICC= 0.77), Equation 2 (ICC= 0.76), and Ramirez-Torun equation (ICC= 0.75). The ICC was low between hydrodensitometry and Durnin-Womersley (ICC= 0.51) and Jackson-Pollock (ICC= 0.53) equations.

**Conclusion:**

The new Equations-1 and 2, performed better than the commonly used anthropometric equations to predict BF% in adult women.

## Resumen

**Objetivo:**

Desarrollar ecuaciones antropométricas para predecir el porcentaje de grasa corporal (%GC).

**Métodos:**

En 151 mujeres **(**18-59 años) se midieron peso corporal, estatura, ocho pliegues cutáneos (PCs), seis perímetros (PEs) y el %GC por hidrodensitometría. Se formaron dos grupos al azar, desarrollo de ecuaciones (n=106) y validación (n= 45). En el grupo desarrollo ecuaciones se calcularon modelos de regresión lineal, con las medidas antropométricas como predictores, para encontrar la mejor ecuación de predicción del %GC. El grupo validación se utilizó para comparar el desempeño de las nuevas ecuaciones con las de Durnin-Womersley, Jackson-Pollock y Ramírez-Torun.

**Resultados:**

Se seleccionaron dos ecuaciones: Ecuación-1= 11.76 + (0.324 x tríceps PC) + (0.133 x pantorrilla-medial PC) + (0.347 x abdomen PE) + (0.068 x edad-años) - (0.135 x estatura) y Ecuación-2= 11.37 + (0.404 x tríceps PC) + (0.153 x axilar PC) + (0.264 x abdomen PE) + (0.069 x edad-años) - (0.099 x estatura). No hubo diferencias significativas en el %GC obtenido por hidrodensitometría (31.5 ±5.3) y Ecuación-1 (31.0 ±4.0) o Ecuación-2 (31.2 ±4.0). Los %GC estimados por Durning-Womersley (35.8 ±4.0), Jackson-Pollock (26.5 ±5.4) y Ramírez-Torun (32.6 ±4.8) fueron diferentes del obtenido por hidrodensitometría (*p* <0.05). El coeficiente de correlación intraclase (ICC) fue alto entre hidrodensitometría y las Ecuaciones 1 (ICC= 0.77), 2 (ICC= 0.76), y Ramírez-Torun (ICC= 0.75). El ICC fue bajo entre hidrodensitometría y Durnin-Womersley (ICC= 0.51) y Jackson-Pollock (ICC= 0.53).

**Conclusión:**

Las nuevas ecuaciones 1 y 2 presentaron mejor rendimiento que las ecuaciones tradicionales para predecir el %GC en mujeres adultas.

## Introduction

Colombian adult women (age range 18 to 64 years) had a high prevalence of overweight (35.0%) and obesity (20.1%) that coexists with less proportion of underweight people (3.0%) ^1^. These prevalences are based on the body mass index (BMI) application ^1^. The BMI is a body weight-height index that does not differentiate the fat mass from the fat free mass ^2^ ^,^ ^3^. This is relevant since it is the excess of fat mass, nor necessarily the excess of body weight, that represents higher risk of developing cardiovascular diseases and type 2 diabetes ^2^ ^,^ ^4^. Correspondingly, is the deficit of fat free mass, frequently observed in underweight, but also in normal weight and overweight people, that associates with negative clinical outcomes, lower functional capacities, and impairment of overall health ^3^. Therefore, in sceneries looking to prevent, diagnose, and treat underweight, overweight, and obesity, the application of methods to assess body composition (i.e. fat mass and fat free mass) are preferred than the body weight-height indices like BMI ^2^ ^,^ ^3^.

Anthropometric equations are widely used to estimate body composition and recently new equations have been developed for specific populations ^5^ ^-^ ^9^. The equations are developed following three general steps ^10^. First, body composition is determined in a group of people by a reference method (i.e. a highly acute laboratory method). Second, in the same group of people measurements such as body weight, height, skinfold thickness and circumferences are collected. Third, the collected body measurements are used as predictors to obtain the best equation estimating the quantities of fat mass or fat free mass ^10^. In general, anthropometric equations are population specific, given that the relation between body measurements and body components (i.e. fat mass and fat free mass calculated from body density) are modified by age, sex, and ethnicity ^2^ ^,^ ^11^. Therefore, an anthropometric equation should not be applied to a population different from it was derivate without a previous validation ^2^ ^,^ ^11^. Durning-Womersly ^12^ and Jackson-Pollock ^13^ are traditional equations commonly used to evaluate body composition worldwide. These equations and the more recently published by Ramirez-Torun ^14^ have shown poor validity to estimate body composition in Colombian women ^15^.

This study aimed to develop and validate practical anthropometric equations to estimate body composition in women living in Medellín-Colombia. The study hypothesized the new equations will perform better in Colombian women than the equations developed in foreign countries.

## Materials and Methods

### Study design and participants

This is a cross-sectional study with a convenience sample of 151 women with ages between 18 to 59 years. Participants were students, teachers and volunteers attending the outreach programs from the University of Antioquia, Medellín-Colombia. No athletes were included in the study or women with implants (e.g. silicon, plastic, metal), in pregnancy or having any other physiological conditions that might have altered the results. The study was approved by the Bioethics Committee of the Faculty of Medicine from the University of Antioquia and was performed according to the Helsinki Declaration. Written consent was obtained from each participant. 

Hydrodensitometry and anthropometric measurements were done in the Human Body Composition Laboratory, at the School of Nutrition and Dietetics from the University of Antioquia, Medellín-Colombia. Participants were scheduled to attend the laboratory a day that did not include five days before or after menses. Volunteers were asked to avoid intense physical activity and food that produces gases (e.g. beans, broccoli, and cabbage) the day before the test. Participants arrived at the laboratory between 7:00 am and 9:00 am after a fast period of at least four hours. After urinating/defecating volunteers removed garments, jewels, and wore a bathing suit for anthropometric and hydrodensitometry measurements.

### Anthropometry

Measurements were carried out by two trained anthropometrists following the standard techniques described by Lohman, *et al* ^16^. Body weight was measured to the nearest 0.05 kg using a digital scale (Detecto CN20LS, USA). Height was measured to the nearest 0.1 cm using an anthropometer (GPM 101, Switzerland). Arm, waist, abdominal, hip, thigh, and calf circumferences were measured to the nearest 0.1 cm using a metal tape (Lufkin W606PM, USA). Skinfold thickness was measured to the nearest 0.2 mm with a caliper (Harpenden CE0120, England) including biceps, triceps, subscapular, midaxillary, suprailiac, abdominal, medium thigh, and medial calf. Anthropometric measurements were done at least by duplicate or by triplicate when the difference between the first and the second values were higher than 0.05 kg in body weight, 0.5 cm in height, 1% in circumferences, and 5% in skinfold thickness. Body composition was calculated using Durning-Womersly ^12^, Jackson-Pollock ^13^, and Ramirez-Torun ^14^ equations.

### Hydrodensitometry

Body density was determined by underwater weighing with simultaneous measurement of residual lung volume. Volunteers entered to a tank filled with water at 36 ±0.2^º^C and sat on a plastic chair suspended from a scale with 0.02 kg of sensitivity (Chatillon, C-103616, USA). Participants submerged completely in water using a nose clip and breathing through a mouthpiece connected to a spirometer (Sensor Medics, VMAX 22, USA). Residual lung volume and underwater weight were recorded simultaneously at the end of a maximal exhalation. Body volume was calculated by subtracting underwater body weight (UBW) from body weight, and dividing the difference by water density at 36^º^ C [i.e. body volume = (body weight - UBW) / water density]. Then, body volume was adjusted by subtraction of the residual lung volume and 0.1 L of estimated intestinal gas, as recommended ^17^. Body density was calculated dividing body weight by the adjusted body volume. The whole procedure was repeated at least twice or up to obtain two body densities with a difference ≤ 0.002 g/mL in each participant. The selected body densities were averaged and the BF% was calculated with the Siri equation, BF% = 4.95 / body density - 4.50 ^17^.

### Statistical analysis

Normal distribution of data was tested with the Kolmogorov-Smirnov test. Means, standard deviation and range were calculated for all variables. Participants´ data were randomly divided in two groups; equation-building group (n=106) and validation group (n= 45). Multiple linear regression models were ran using anthropometric data in the equation-building group as predictors, to identify the best prediction equations of the BF%. The equations were ascertained by identifying the models that meet the normality, collinearity, variance homogeneity and the Durbin Watson´s criterion. The Akaike information criterion (AIC) that estimates the quality of the statistical models was also calculated for each equation. Two selected equations using two skinfold thickness, one circumference, height, and age, showed a good adjusted determination coefficient (adjusted r^2^) and a low standard error of the estimate (SEE). Using the same criteria, a third equation that did not include skinfold thickness among the predictors was also selected. These equations were used to estimate the BF% in the validation group and the adjusted r^2^ and the SEE were obtained. Averages of BF%, fat mass, and fat free mass estimated by the new equations and those of Durning-Womersley, Jackson-Pollock and Ramirez-Torun were compared with hydrodensitometry using paired t-test. Pearson correlation coefficient and Intraclass correlation coefficient (ICC) for BF% were also calculated. Data analyses were made using The Statistical Package for Social Sciences for Windows (SPSS. 22.0, 2013, SPSS, Inc, Chicago, IL).

## Results

Twelve subjects did not successfully complete the underwater weighing test, mainly for being unable to breathe underwater through the mouthpiece. These subjects were excluded from the analysis and did no differ in any anthropometric measurements from the participants used to develop and validate the equations. Complete anthropometric measurements and underweight weighing were obtained in 151 women, ranging from 18 to 59 years old. Participants´ data were randomly divided in two groups, equation-building group (n= 106) and validation group (n= 45). There were not significant differences between groups in age (33.5 ±12.9; 35.0 ±11.9 y, *p*= 0.656), BMI (23.6 ±3.0; 23.7 ±3.4 kg/m^2^, *p*= 0.833), BF% (31.2 ±5.9; 31.3 ±6.1, *p*= 0.975) or any anthropometric measurement (Table 1). The BF% ranged between 19% to 44% in the equation-building group, and between 21% to 44% in the validation group (Table 1). 

**Table 1:** Subject characteristics by group

Characteristics	Equation-building group (n= 106)		Validation group (n= 45)		*p*-value
	Mean ± SD	Range	Mean ± SD	Range	
Age (yrs)*	33.5 ± 12.9	18-59	35.0 ± 11.9	19-59	0.656
Body weight (kg)*	58.6 ± 8.0	42-83	59.6 ± 8.2	43-72	0.669
Height (cm)	157.6 ± 6.5	143-175	158.5 ± 6.4	143-174	0.309
Body mass index (kg/m^2^)*	23.6 ± 3.0	18-31	23.7 ± 3.4	19-32	0.833
Arm circumference (cm)*	27.7 ± 2.6	22-34	28.2 ± 3.1	23-36	0.333
Waist circumference (cm)*	*74.4 ± 7.8*	59-93	74.5 ± 8.4	61-89	0.851
Abdominal circumference (cm)*	84.2 ± 7.6	69-105	85.4 ± 6.9	69-101	0.528
Hip circumference (cm)	97.5 ± 5.6	84-105	97.9 ± 5.7	84-112	0.726
Medium-thigh circumference (cm)	49.4 ± 4.0	41-60	49.9 ± 3.6	41-58	0.456
Calf circumference (cm)	35.3 ± 2.5	30-44	35.8 ± 2.5	31-42	0.391
Bicipital skinfold (mm)*	10.2 ± 3.4	4-25	10.5 ± 4.7	4-17	0.372
Tricipital skinfold (mm)	19.5 ± 4.9	11-32	21.3 ± 5.7	9-30	0.072
Subscapular skinfold (mm)*	22.5 ± 8.8	8-47	22.9 ± 9.1	8-50	0.085
Midaxillary skinfold (mm)*	17.7 ± 7.0	7-38	18.5 ± 7.6	7-36	0.801
Suprailiac skinfold (mm)	34.0 ± 8.0	18-52	34.8 ± 7.6	12-52	0.187
Abdominal skinfold (mm)*	28.2 ± 7.8	14-59	27.4 ± 7.1	14-51	0.723
Medium-thigh skinfold (mm)	27.1 ± 7.9	11-48	29.7 ± 9.8	13-51	0.092
Medial-calf skinfold (mm)	19.5 ± 6.4	6-33	19.8 ± 7.9	5-43	0.866
Body density (g/mL)	1.028 ± 0.012	1,003-1,055	1.029 ± 0.013	1,005-1,055	0.820
Body Fat (%)	31.2 ± 5.9	19-44	31.3 ± 6.1	21-44	0.975

Differences between groups were calculated by T-test.

*In non-normally distributed variables the Mann-Whitney U test was used.

The selected anthropometric equations for estimating BF% are showed in Table 2. Equation 1 includes the measurements of body height, abdominal circumference, triceps- and calf- skinfold thickness plus age. Equation 2 includes the same measurements than Equation 1 except for the calf skinfold that was replaced by the midaxillary skinfold. Equation-3 included body weight, height and abdominal circumference measurements. Equations 1 and 2 had similar determination coefficients and SEE in the equation-building group (Table 2). Equation 1 showed a slightly better performance than Equation 2 during the validation process with a higher determination coefficient (0.71 Vs 0.67) and lower SEE (2.84 Vs 3.06). Equation 3 had lower determination coefficient and higher SEE than Equations 1 and 2, in both, equation-building and validation group (Table 2). The Akaike information criterion was similar between Equation 1 and 2 (549 vs 547). The Equation 3 had higher AIC than Equations 1 and 2 (AIC= 569; *p* <0.001) presenting a lower quality statistical model (Table 2).

**Table 2:** Developed anthropometrics equations to predict body fat percentage

**Equations**	**Equation-building group** ^**§**^			**Validation group**	
	**Adjusted r** ^**2**^	**SEE**	**AIC**	**r** ^**2**^	**SEE**
**1:** Body fat (%) = 11.76 + (0,324 x triceps ST) + (0.133 x calf ST) + (0.347 x abdomen CI) + (0.068 x age in years) - (0.135 x height in cm.)	0.72	3.12	549	0.71	2.84
**2:** Body fat (%) = 11.37 + (0.404 x triceps ST) + (0.153 x midaxillary ST) + (0.264 x abdomen CI) + (0.069 x age in years) - (0.099 x height in cm.)	0.72	3.08	547	0.67	3.06
**3:** Body fat (%) = 27.39 + (0.264 x body weight in kg) + (0.381 x abdomen CI) - (0.279 x height in cm)	0.66	3.44	569*	0.55	3.55

SEE: Standard error of estimate. AIC: Akaike Information Criterium. r^2^: determination coefficient. ST: skinfold thickness in mm, CI: circumference in cm.

*Different from Equation 1 and Equation 2 (*p* <0.001).

^§^Assumption models (p value).

Equation 1: Shapiro Wilk test= 0.9543; Durbin-Watson test= 0.9023; Homogeneity of variances test= 0.4803; Variance Inflate Factor <2.8.

Equation 2: Shapiro Wilk test= 0.1318; Durbin-Watson test= 0.9535; Homogeneity of variances test=0.8445; Variance Inflate Factor <3.4.

Equation 3: Shapiro Wilk test= 0.1489; Durbin-Watson test= 0.8721; Homogeneity of variances test= 0.5135; Variance Inflate Factor <4.5.

The BF% obtained by hydrodensitometry and anthropometric equations in the validation group are shown in Table 3. There were not significant differences (*p* >0.05) between the BF% assessed by hydrodensitometry (31.5 ±5.3) and the estimated by Equation 1 (31.0 ±4.0), Equation 2 (31.2 ±4.0) and Equation 3 (31.0 ±4.6). The BF% was over estimated by the equations of Durning-Womerley (+4.26; *p* <0.001) and Ramirez-Torun (+1.10; *p* <0.05) and underestimated by the equation of Jackson-Pollock (-5.03; *p* <0.001) (Table 3). The BF% estimated by the anthropometric equations significantly correlated (*p* <0.001) with the hydrodensitometry results, the higher correlations were observed for Equation 1 (r= 0.81; *p* <0.001, ICC= 0.77; *p* <0.001) and Equation 2 (r= 0.79; *p* <0.001, ICC= 0.76; *p* <0.001) (Table 3).

**Table 3:** Comparison of body fat percentage obtained by hydrodensitometry and anthropometric equations.

Validation group (n= 45)	Body fat (%)	Differences from Hydro†	Pearson correlation	Intraclass correlation
Hydrodensitometry	31.5 ± 5.3	---	---	---
Equation 1	31.0 ± 4.0	0.50	0.81**	0.77**
Equation 2	31.2 ± 4.0	0.31	0.79**	0.76**
Equation 3	31.0 ± 4.6	0.49	0.74**	0.73**
Durnin-Womersley	35.8 ± 4.0	4.26**	0.75**	0.51**
Jackson-Pollock	26.5 ± 5.4	-5.03**	0.77**	0.53**
Ramirez-Torun	32.6 ± 4.8	1.10*	0.77**	0.75**

**†**Differences from hydrodensitometry calculated by paired T-test.

*p <0.05. **p <0.001.

Body composition obtained by hydrodensitometry and anthropometric equations in the validation group are shown in Table 4. There were not significant differences (*p* >0.05) between the kilograms of fat mass and fat free mass obtained by hydrodensitometry (19.0 ±4.9; 40.5 ±4.2, respectively) and those estimated by Equation 1 (18.7 ±4.4; 40.8 ±3.7, respectively), Equation 2 (18.8 ±4.4; 40.7 ±3.6, respectively) and Equation 3 (18.7 ±4.8; 40.7±3.4, respectively). The equations of Durning-Womerley, Jackson-Pollock and Ramirez-Torun estimated quantities of fat mass and fat free mass significantly different (*p* <0.05) from those obtained by the reference method (Table 4).

**Table 4:** Comparison of body fat mass and fat free mass obtained by hydrodensitometry and anthropometric equations.

Validation group (n= 45)	Fat Mass (kg)		Fat Free Mass (kg)	
	Mean (±SD)	Diff. from Hydro†	Mean (±SD)	Diff. from Hydro†
Hydrodensitometry	19.0 ± 4.9	---	40.5 ± 4.2	---
Equation 1	18.7 ± 4.4	-0.30	40.8 ± 3.7	0.30
Equation 2	18.8 ± 4.4	-0.18	40.7 ± 3.6	0.18
Equation 3	18.7 ± 4.8	-0.24	40.7 ± 3.4	0.23
Durnin-Womersley	21.5 ± 4.5	2.50**	38.0 ± 3.7	-2.50**
Jackson-Pollock	16.0 ± 4.8	-2.96**	43.4 ± 4.2	2.96**
Ramirez-Torun	19.7 ± 5.1	0.72*	39.7 ± 3.1	-0.73*

**†**Differences from hydrodensitometry calculated by paired T-test.

*p <0.05. **p <0.001.

## Discussion 

The objective of this study was to develop and validate anthropometric equations to estimate body composition in adult women. Two equations including measurements of body height, abdominal circumference, and two skinfolds thickness were developed and validated. These equations are advised to be applied in clinical settings. A third equation without skinfold thickness measurement was also developed and it is suggested to be use in epidemiological settings. The three equations meet the criteria that good anthropometric equations should have according to Heyward and Stolarczyk ^18^, as follows: a) the use of an acceptable reference method like hydrodensitometry, b) the use of a large sample size higher than 100 subjects, c) to show high multiple correlations between the reference method and the predicted scores, d) to have a small SEE, and e) to be cross-validated on additional, independent sample from the population ^18^.

The developed anthropometric equations meet statistical and practicality criteria. Multiple anthropometric equations were identified by combining age with the sixteen body measurements collected (Table 1). First, the equations were ascertained by identifying the models that meet the statistical criteria (see statistical analysis section). Then, looking for practical equations, those that included lower body measurements were selected. Equations 1 and 2 used the same number of measurements, but Equation 1 is the first option since includes skinfold measurement at triceps and calf, sites that are easier to access than the midaxillary skinfold required for Equation 2. In addition, Equation 1 showed a slightly better performance than Equation 2 in the validation group. Nonetheless, both equation are classified as very good, according to the Lohman´s classification system to evaluate new methods, given their SSE of 3.12 and 3.08, respectively ^19^. Equations 1 and 2 explained 72% of the variance in BF%, probably due to the lower number of body measurements they included; two skinfolds and one circumference. This theory is supported by the findings of Ramirez-Torun who developed equations in Guatemalan women; they reported that models including three skinfolds and two circumferences explain up to 88% of the variance in BF%, and equations with lower body measurements explained lower variances ^14^. Equation 3 is the easiest to be applied since it uses only three body measurements and does not include skinfold thickness. Although this equation with an SEE of 3.44 is classified as good according to the Lohman´s classification system ^19^ its performance in the validation group was lower than Equations 1 and 2, and it had also a lower quality statistical model. The Equation 3 was design to be applied in epidemiological settings where it is harder to have trained anthropometrics for skinfold measurements and to maintain good quality- calibrated calipers.

The developed anthropometric equations performed better than the foreign equations estimating body composition in adult Colombian women. The Equations 1, 2 and 3 produced body composition results similar to hydrodensitometry, and they showed substantial correlation and agreement with this reference method according to Landis criteria ^20^. Contrarily, the equations of Durning-Womersley, Jackson-Pollock and Ramirez-Torun showed significant differences with the reference method, results in agreement with previous studies ^9^ ^,^ ^14^ ^,^ ^15^ ^,^ ^21^ ^-^ ^23^. The poor results of the foreign equations could be due to differences among ethnic groups in density of body components, body fat distribution or body segment proportions, characteristics that affect equations´ performance ^9^ ^-^ ^11^. It seems that the equations of Durning-Womersley and Jackson-Pollock developed in Caucasian, and the Ramirez-Torun designed in Guatemalan people (a mixture of European and Amerindian) are not appropriated for Colombian women who are described as a mixture of European, African and Native American ^24^ ^,^ ^25^. 

According to the authors´ knowledge, this study is unique in developing and validating anthropometric equations to estimate BF% in women living in Colombia and using hydrostatic weighing. Strength of this study is the development of an anthropometric equation without skinfold thickness measurement, which is suggested to be applied in epidemiological settings where skinfold thickness is not commonly measured. The study has some limitations. The use of a convenience sample, given that hydrostatic weighing is a demanding test where voluntaries remain underwater during 2 to 4 minutes breathing through a mouthpiece. This makes the reference test unfeasible for representative population samples. Therefore, careful interpretation of the results from the new equations is recommended when they are applied to general population, particularly to individuals who significantly differ from the participants of this study; differences in anthropometric measurements, ethnicity and physical activity level should be especially considered. Another study limitation is the use of a two-compartmental model that assumes constant densities of the fat mass and the fat free mass; it is known that there are variations in the density of these components with age, ethnicity and fitness level ^17^. The use of a four-compartmental model would overcome this difficulty but it was not applied in this study due to unavailability of resources. It is important to highlight that the Equations 1, 2 and 3 showed good performance in the validation group with averages of BMI and BF% of 23.7 kg/m^2^ and 31.3%, respectively. Future studies should validate these equations in groups of women with wider BMI and fat mass distribution. 

In summary, the new developed Equations 1 and 2 performed better than the commonly used anthropometric equations to predict body composition in women from Medellin-Colombia. The Equation 3 has a higher SEE and lower quality statistical model than Equations 1 and 2, and it was design to be applied in epidemiological settings. Careful interpretation of the equations´ results is recommended when they are applied to individuals with physical characteristics that significantly differ from the participants of this study.
