# Supplementary material for: Development and validation of anthropometric equations to estimate body composition in adult women
Source: Colomb Med (Cali). 2018 Jun 30;49(2):154–9. doi: 10.25100/cm.v49i2.3643 (PMC6084924; doi:10.25100/cm.v49i2.3643)
Supplement: Related texts

# Research article: Desarrollo y validación de ecuaciones antropométricas para estimar la composición corporal en mujeres adultas

## Introducción

Las mujeres adultas colombianas (18 a 64 años) tienen una alta prevalencia de sobrepeso (35.0%) y obesidad (20.1%) que coexiste con una menor proporción de personas con bajo peso (3.0%) ^1^. Estas prevalencias están basadas en la aplicación del índice de masa corporal (IMC) ^1^. El IMC es un índice que no puede diferenciar la cantidad de masa grasa de la masa libre de grasa ^2^^,^^3^. Esto es relevante porque es el exceso de masa grasa, no necesariamente el exceso de peso corporal, lo que representa un mayor riesgo para desarrollar enfermedades cardiovasculares y diabetes tipo 2 ^2^^,^^4^. De forma similar, es el déficit de masa libre de grasa, observado en las personas con bajo peso pero también en las de peso normal y en sobrepeso, el que se asocia a resultados clínicos negativos, baja capacidad funcional y deterioro de la salud en general ^3^. Por lo tanto, en escenarios de prevención, diagnóstico y tratamiento del bajo peso, el sobrepeso y la obesidad, se prefiere la evaluación de la composición corporal (masa grasa y masa libre de grasa) que la sola aplicación del IMC ^2^^,^^3^.

Las ecuaciones antropométricas son ampliamente usadas para estimar la composición corporal y, recientemente, nuevas ecuaciones han sido desarrolladas para poblaciones específicas ^5^^-^^9^. Las ecuaciones son desarrolladas generalmente en tres pasos ^10^. Primero, la composición corporal es determinada en un grupo de personas por un método de referencia, estos métodos son de alta precisión y, por lo general, son complejos y se realizan en laboratorios. Segundo, en el mismo grupo de personas se hacen medidas antropométricas como peso corporal, estatura, pliegues cutáneos y perímetros corporales. Tercero, las medidas antropométricas recolectadas son usadas como predictores para obtener la mejor ecuación que estime la cantidad de masa grasa o masa libre de grasa ^10^. En general, las ecuaciones antropométricas son específicas de población, dado que la relación entre las medidas corporales y los componentes (masa grasa y masa libre calculados a partir de la densidad corporal) es modificada por la edad, el sexo y el grupo étnico ^2^^,^^11^. Por lo tanto, las ecuaciones antropométricas no deberían ser utilizadas en poblaciones diferentes de las cuales fueron derivadas, sin una previa validación ^2^^,^^11^. Las ecuaciones de Durning-Womersly ^12^ y Jackson-Pollock ^13^ son las más comúnmente utilizadas para evaluar la composición corporal. Estas ecuaciones y la más reciente publicada por Ramírez-Torun ^14^ han mostrado una pobre validez para estimar la composición corporal en mujeres colombianas ^15^.

El objetivo de este estudio fue desarrollar y validar ecuaciones antropométricas prácticas para estimar la composición corporal en mujeres que viven en Medellín-Colombia. La hipótesis del estudio es que las nuevas ecuaciones tendrán un mejor desempeño en mujeres colombianas que las ecuaciones desarrolladas en países foráneos.

## Materiales y métodos

### Diseño del estudio y participantes

Estudio transversal con una muestra a conveniencia de 151 mujeres con edades entre 18 y 59 años. Las participantes fueron estudiantes, profesoras y personas que asisten a los programas de extensión de la Universidad de Antioquia, Medellín-Colombia. Fueron excluidas mujeres atletas, con implantes de silicona, plástico o metal, embarazadas o que tuvieran alguna condición fisiológica que pudiera alterar los resultados. El estudio fue aprobado por del Comité de Bioética de la Facultad de Medicina de la Universidad de Antioquia y realizado de acuerdo a la Declaración de Helsinki. Se obtuvo el consentimiento informado de todas las participantes.

Las pruebas de antropometría e hidrodensitometría fueron realizadas en el Laboratorio de Composición Corporal Humana de la Escuela de Nutrición y Dietética de la Universidad de Antioquia, Medellín-Colombia. Se programó a cada participante para que el día de las pruebas no estuviera entre los cinco días previos o posteriores al periodo menstrual. Se solicitó a las voluntarias no realizar actividad física moderada-intensa o consumir alimentos productores de gases (por ejemplo, frijoles, brócoli, repollo, etc.) el día antes de la prueba. Las participantes ingresaron al laboratorio entre las 7:00 a.m. y las 9:00 a.m. con un periodo de ayuno mayor a cuatro horas. Después de haber eliminado orina y materia fecal, las voluntarias se despojaron de joyas y accesorios, y se colocaron un traje de baño de dos piezas.

### Antropometría

Las mediciones fueron realizadas por dos antropometristas entrenados y siguiendo las técnicas descritas por Lohman, *et al* ^16^. El peso corporal fue medido con una báscula digital (Detecto CN20LS, USA) con una precisión de 0.05 kg. La estatura con un antropómetro (GPM 101, Suiza), con una precisión de 0.1 cm. Los perímetros de brazo, cintura, abdomen, cadera, muslo medio y pantorrilla fueron medidos con una cinta métrica (Lufkin W606PM, USA) con una precisión de 0.1 cm. Los pliegues cutáneos fueron medidos con un calibrador (Harpenden CE0120, Inglaterra) con una precisión de 0.2 mm. Se midieron los pliegues bíceps, tríceps, subescapular, axilar, suprailiaco, abdominal, muslo medio y pantorrilla medial. Las mediciones fueron hechas por duplicado o por triplicado cuando la diferencia entre la primera y la segunda medición fue mayor a 0.05 kg en el peso corporal, 0.5 cm en la estatura, 1% en los perímetros y 5% en los pliegues cutáneos. La composición corporal fue calculada con las ecuaciones de Durning-Womersly ^12^, Jackson-Pollock ^13^ y Ramírez-Torun ^14^.

### Hidrodensitometría

La densidad corporal fue determinada por pesaje bajo el agua con la medición simultánea del volumen residual pulmonar. Las voluntarias ingresaron a un tanque de fibra de vidrio lleno de agua a 36±0.2° C y permanecieron sentadas sobre un silla plástica suspendida de una báscula (Chatillon, C-103616, USA) de 0.02 kg de sensibilidad. Las participantes se sumergieron completamente en el agua con la nariz tapada con un clip nasal y utilizando una boquilla para respirar a través de un espirometro (Sensor Medics, VMAX 22, USA). El volumen residual y el peso bajo el agua fueron registrados de manera simultánea al final de una expiración máxima. El volumen corporal fue calculado restando del peso corporal el peso bajo el agua (PBA) y dividiendo esta diferencia por la densidad del agua a 36° C [volumen corporal= (peso corporal - PBA) / densidad del agua]. Luego, el volumen corporal fue ajustado con la sustracción del volumen residual pulmonar y el volumen del gas intestinal (0.1 L valor promedio) como es recomendado ^17^. La densidad corporal fue calculada dividiendo el peso corporal por el volumen corporal ajustado. Todo el proceso fue repetido, dos veces o más en cada participante, hasta obtener mediciones de la densidad corporal con una diferencia ≤0.002 g/mL. Los valores de la densidad corporal que se encontraban en la diferencia permitida fueron promediados para calcular el %GC con la ecuación de Siri, %GC= 4.95/densidad corporal -4.50 ^17^.

## Análisis estadístico

Se evaluó la normalidad de los datos con la prueba de Kolmogorov-Smirnov. El promedio y la desviación estándar fueron calculados para cada variable. Los datos de las participantes fueron asignados de manera aleatoria a uno de dos grupos: grupo para desarrollo de ecuaciones (n= 106) y grupo de validación (n= 45). En el grupo de desarrollo de ecuaciones se calcularon modelos de regresión lineal con las medidas antropométricas como predictores para encontrar la mejor ecuación de predicción del %GC. Se seleccionaron los modelos de predicción que cumplieran los criterios de normalidad, colinealidad, homegenidad de varianza y el criterio de Durbin-Watson. El criterio de información de Akaike (AIC) que estima la calidad de los modelos estadísticos, fue calculado para cada ecuación. Dos de las ecuaciones seleccionadas, las cuales utilizan dos pliegues cutáneos, un perímetro, la estatura y la edad, presentaron un coeficiente de determinación ajustado (r^2^ ajustado) adecuado y un error estándar de estimación (SEE) bajo. Usando los mismos criterios de selección una tercera ecuación, que no incluyó pliegues cutáneos en el modelo de predicción, fue también seleccionada. Estas ecuaciones fueron utilizadas para estimar el %GC en el grupo de validación y se calculó el valor de r^2^ ajustado y el SEE. El promedio del %GC, la masa grasa y la masa libre de grasa estimado por las nuevas ecuaciones y las de Durning-Womersley, Jackson-Pollock y Ramírez-Torun fueron comparados con el obtenido por hidrodensitometría utilizando la prueba t. Se calculó el coeficiente de correlación de Pearson y el ICC para el %GC. El análisis de los datos se hizo con el paquete estadístico Statistical Package for Social Sciences for Windows (SPSS 22.0, 2013, SPSS, Inc, Chicago, IL).

## Resultados

Doce sujetos no completaron la prueba de pesaje hidrostático, principalmente por no ser capaces de respirar bajo el agua a través de la boquilla. Estos sujetos fueron excluidos del análisis y no se diferenciaron, en ninguna medida antropométrica, de los individuos que conformaron los grupos para el desarrollo y la validación de las ecuaciones. Las mediciones antropométricas completas y el pesaje bajo el agua fueron obtenidos para 151 mujeres, de 18 a 59 años. Los datos de las participantes fueron divididos aleatoriamente en dos grupos, grupo de desarrollo de ecuaciones (n= 106) y grupo de validación (n= 45). No se observaron diferencias significativas entre los grupos en edad (33.5 ±12.9; 35.0 ±11.9 años, *p*= 0.656), IMC (23.6 ±3.0; 23.7 ±3.4 kg/m^2^, *p* = 0.833), %GC (31.2 ±5.9; 31.3 ±6.1, *p*= 0.975) u otras variables (Tabla 1). El rango de %GC estuvo entre 19% y 44% en el grupo de desarrollo de ecuaciones, y entre 21% y 44% en el grupo de validación (Tabla 1).

**Tabla 1:** Características de los sujetos por grupos

Characteristicas	Grupo desarrollo (n= 106)		Grupo validación (n= 45)		valor *p*
	Media ±DE	Rango	Media ±DE	Rango	
Edad (años)*	33.5 ± 12.9	18-59	35.0 ± 11.9	19-59	0.656
Peso (kg)*	58.6 ± 8.0	42-83	59.6 ± 8.2	43-72	0.669
Estatura (cm)	157.6 ± 6.5	143-175	158.5 ± 6.4	143-174	0.309
Índice de masa corporal (kg/m^2^)*	23.6 ± 3.0	18-31	23.7 ± 3.4	19-32	0.833
Perímetro brazo medio (cm)*	27.7 ± 2.6	22-34	28.2 ± 3.1	23-36	0.333
Perímetro cintura (cm)*	*74.4 ± 7.8*	59-93	74.5 ± 8.4	61-89	0.851
Perímetro abdomen (cm)*	84.2 ± 7.6	69-105	85.4 ± 6.9	69-101	0.528
Perímetro cadera (cm)	97.5 ± 5.6	84-105	97.9 ± 5.7	84-112	0.726
Perímetro muslo medio (cm)	49.4 ± 4.0	41-60	49.9 ± 3.6	41-58	0.456
Perímetro pantorrilla (cm)	35.3 ± 2.5	30-44	35.8 ± 2.5	31-42	0.391
Pliegue bíceps (mm)*	10.2 ± 3.4	4-25	10.5 ± 4.7	4-17	0.372
Pliegue tríceps (mm)	19.5 ± 4.9	11-32	21.3 ± 5.7	9-30	0.072
Pliegue subscapular (mm)*	22.5 ± 8.8	8-47	22.9 ± 9.1	8-50	0.085
Pliegue axilar (mm)*	17.7 ± 7.0	7-38	18.5 ± 7.6	7-36	0.801
Pliegue suprailiaco (mm)	34.0 ± 8.0	18-52	34.8 ± 7.6	12-52	0.187
Pliegue abdominal (mm)*	28.2 ± 7.8	14-59	27.4 ± 7.1	14-51	0.723
Pliegue muslo medio (mm)	27.1 ± 7.9	11-48	29.7 ± 9.8	13-51	0.092
Pliegue pantorrilla medial (mm)	19.5 ± 6.4	6-33	19.8 ± 7.9	5-43	0.866
Densidad corporal (g/mL)	1.028 ± 0.012	1,003-1,055	1.029 ± 0.013	1,005-1,055	0.820
Porcentaje de grasa corporal (%)	31.2 ± 5.9	19-44	31.3 ± 6.1	21-44	0.975

Los valores son promedio ± desviación estándar y rango en paréntesis (Min = mínimo, Max. = máximo). Las diferencias entre grupos fueron calculadas por la prueba t.

*Para las variables que no se distribuyeron de forma normal se aplicó la prueba U de Mann-Whitney.

Las ecuaciones antropométricas seleccionadas para estimar el %GC se muestran en la Tabla 2. La Ecuación 1 incluye las mediciones de estatura, perímetro de abdomen, pliegues cutáneos de tríceps y pantorrilla medial, y la edad. La Ecuación 2 incluye las mismas medidas de la ecuación 1 excepto el pliegue pantorrilla medial que fue remplazado por el pliegue axilar. La Ecuación 3 incluyó el peso corporal, la estatura y el perímetro de abdomen. Las Ecuaciones 1 y 2 tuvieron un coeficiente de determinación y un SEE similar en el grupo de desarrollo de ecuaciones (Tabla 2). La Ecuación 1 mostró un desempeño ligeramente superior que la Ecuación 2 en el grupo de validación, con un coeficiente de determinación más alto (0.71 Vs 0.67) y un SEE más bajo (2.84 Vs 3.06). La Ecuación 3 presentó el coeficiente de determinación más bajo y el SEE más alto, en comparación con las Ecuaciones 1 y 2, en ambos grupos, desarrollo y validación (Tabla 2). El criterio de información de Akaike fue similar en las Ecuaciones 1 y 2 (549 Vs 547). La Ecuación 3 tuvo un criterio de información mayor que las ecuaciones 1 y 2 (AIC= 569; *p* <0.001) mostrando una inferior calidad del modelo estadístico (Tabla 2).

**Tabla 2:** Ecuaciones antropométricas desarrolladas para predecir el porcentaje de grasa corporal

	**Grupo desarrollo** ^**§**^			**Grupo validación**	
**Ecuaciones**	**r** ^**2**^ **ajustado**	**SEE**	**AIC**	**r** ^**2**^ **ajustado**	**SEE**
**1:** Porcentaje de grasa (%) = 11.76 + (0,324 x PC tríceps) + (0.133 x PC pantorrilla medial) + (0.347 x PE abdomen) + (0.068 x edad en años) - (0.135 x estatura en cm)	0.72	3.12	549	0.71	2.84
**2:** Porcentaje de grasa (%) = 11.37 + (0.404 x PC tríceps) + (0.153 x PC axilar) + (0.264 x PE abdomen) + (0.069 x edad en años) - (0.099 x estatura en cm)	0.72	3.08	547	0.67	3.06
**3:** Porcentaje de grasa (%) = 27.39 + (0.264 x Peso en kg.) + (0.381 x PE abdomen) - (0.279 x estatura en cm)	0.66	3.44	569**	0.55	3.55

SEE: Error estándar del estimado. AIC: Criterio de información de Akaike. r^2^: coeficiente de determinación. PC: pliegue cutáneo en mm, PE: perímetro en cm.

**Diferencia con Ecuación 1 y Ecuación 2 (*p* <0.001).

^§^Supuestos del modelo. Ecuación 1: Prueba de Shapiro Wilk=0.9543; Prueba de Durbin-Watson= 0.9023; Prueba de homogeneidad de la varianza=0.4803; Factor de inflación de la varianza: <2.8. Ecuación 2: Prueba de Shapiro Wilk=0.1318; Prueba de Durbin-Watson= 0.9535; Prueba de homogeneidad de la varianza=0.8445; Factor de inflación de la varianza: <3.4. Ecuación 3: Prueba de Shapiro Wilk=0.1489; Prueba de Durbin-Watson= 0.8721; Prueba de homogeneidad de la varianza=0.5135; Factor de inflación de la varianza: <4.5.

El %GC obtenido por hidrodensitometría y las ecuaciones antropométricas en el grupo de validación se muestran en la Tabla 3. No hubo diferencias significativas (*p* >0.05) entre el %GC medido por hidrodensitometría (31.5 ±5.3) y el estimado por la Ecuación 1 (31.0 ±4.0), Ecuación 2 (31.2 ±4.0) y Ecuación 3 (31.0 ±4.6). Los %GC fueron sobreestimados por Durning-Womersley (+4.26; *p* <0.001) y Ramírez-Torun (+1.10; *p* <0.05) y subestimados por la ecuación de Jackson-Pollock (-5.03; *p* <0.001) (Tabla 3). El %GC estimado por las ecuaciones antropométricas correlacionó con los resultados de la hidrodensitometría, la correlación más alta se observó con la Ecuación 1 (r= 0.81; *p* <0.001, ICC= 0.77; *p* <0.001) y la Ecuación 2 (r= 0.79; *p* <0.001, ICC= 0.76; *p* <0.001) (Tabla 3).

**Tabla 3:** Comparación del porcentaje de grasa corporal obtenido por hidrodensitometría y las ecuaciones antropométricas.

Grupo validación (n=45)	Porcentaje de grasa (%)	Diferencia con Hidro†	Correlación de Pearson	Correlación intraclase
Hidrodensitometría	31.5 ± 5.3	---	---	---
Ecuación 1	31.0 ± 4.0	0.50	0.81**	0.77**
Ecuación 2	31.2 ± 4.0	0.31	0.79**	0.76**
Ecuación 3	31.0 ± 4.6	0.49	0.74**	0.73**
Durnin-Womersley	35.8 ± 4.0	4.26**	0.75**	0.51**
Jackson-Pollock	26.5 ± 5.4	-5.03**	0.77**	0.53**
Ramírez-Torun	32.6 ± 4.8	1.10*	0.77**	0.75**

**†**Diferencias de la hidrodensitometría calculadas por la prueba t.

*p <0.05. **p <0.001.

La composición corporal obtenida por hidrodensitometría y las ecuaciones antropométricas en el grupo de validación se muestran en la Tabla 4. No hubo diferencias significativas (*p* >0.05) en la masa grasa y la masa libre de grasa obtenida por hidrodensitometría (19.0 ±4.9; 40.5 ±4.2, respectivamente) con las estimadas por la Ecuación 1(18.7 ±4.4; 40.8 ±3.7, respectivamente), la Ecuación 2 (18.8 ±4.4; 40.7 ±3.6, respectivamente) y la Ecuación 3 (18.7 ±4.8 kg; 40.7±3.4 kg, respectivamente). Las ecuaciones de Durning-Womerley, Jackson-Pollock y Ramírez-Torun estimaron cantidades de masa grasa y masa libre de grasa diferentes (*p* <0.05) de aquellas obtenidas por el método de referencia (Tabla 4).

**Tabla 4:** Comparación de la masa grasa y la masa libre de grasa obtenida por hidrodensitometría y las ecuaciones antropométricas.

Grupo validación (n=45)	Masa grasa (kg)		Masa libre de grasa (kg)	
	Promedio (±DE)	Dif. con Hidro†	Promedio (±DE)	Dif. con Hidro†
Hidrodensitometría	19.0 ± 4.9	---	40.5 ± 4.2	---
Ecuación 1	18.7 ± 4.4	-0.30	40.8 ± 3.7	0.30
Ecuación 2	18.8 ± 4.4	-0.18	40.7 ± 3.6	0.18
Ecuación 3	18.7 ± 4.8	-0.24	40.7 ± 3.4	0.23
Durnin-Womersley	21.5 ± 4.5	2.50**	38.0 ± 3.7	-2.50**
Jackson-Pollock	16.0 ± 4.8	-2.96**	43.4 ± 4.2	+2.96**
Ramírez-Torun	19.7 ± 5.1	0.72*	39.7 ± 3.1	-0.73*

**†** Diferencias de la hidrodensitometría calculadas por la prueba t.

*p <0.05. **p <0.001.

## Discusión

El objetivo de este estudio fue desarrollar y validar ecuaciones antropométricas para estimar la composición corporal en mujeres adultas. Dos ecuaciones incluyendo mediciones de estatura, perímetro de abdomen y dos pliegues cutáneos fueron desarrolladas y validadas. Se sugiere que estas ecuaciones sean aplicadas en la evaluación individual. Una tercera ecuación, la cual no incluye pliegues cutáneos, fue desarrollada para la evaluación de grupos poblacionales en estudios epidemiológicos. Las tres ecuaciones cumplen los criterios que una buena ecuación antropométrica debe poseer, según Heyward y Stolarczyk ^18^, los cuales son: a) el uso de un método de referencia adecuado para su desarrollo como es la hidrodensitometría, b) el uso de un tamaño de muestra mayor a 100 sujetos, c) las ecuaciones deben mostrar una alta correlación con los resultados del método, d) las ecuaciones deben tener un SEE bajo, y e) las ecuaciones deben tener una validación cruzada en una muestra independiente de la población ^18^.

Las ecuaciones antropométricas seleccionadas cumplen los criterios estadísticos y son prácticas. Se obtuvieron numerosas ecuaciones para estimar el %GC al utilizar la edad y las dieciséis medidas corporales como predictores (Tabla 1). Para seleccionar las ecuaciones primero se escogieron los modelos que cumplían los criterios estadísticos (normalidad, colinealidad, homegenidad de varianza y el criterio de Durbin-Watson). Luego, se valoró la practicidad de las ecuaciones, aquellas que incluyeron el menor número de medidas corporales fueron seleccionadas. Las Ecuaciones 1 y 2 utilizan el mismo número de mediciones, pero la Ecuación 1 es la primera opción dado que incluye pliegues de tríceps y de pantorrilla medial, sitios que son de fácil acceso comparado con el pliegue axilar requerido para la Ecuación 2. Adicionalmente, la Ecuación 1 mostró un comportamiento ligeramente superior que la Ecuación 2 en el grupo de validación. Sin embargo, ambas ecuaciones son clasificadas como muy buenas, de acuerdo al Sistema de clasificación de Lohman para evaluar nuevos métodos, debido a sus SEE de 3.12 y 3.08 respectivamente ^19^. Las Ecuaciones 1 y 2 explican el 72% de la varianza del %GC, probablemente por el bajo número de medidas corporales que incluyen; dos pliegues cutáneos, un perímetro y la estatura. Esta teoría es soportada por los hallazgos de Ramírez-Torun quienes desarrollaron ecuaciones para mujeres guatemaltecas ^14^. Los autores reportaron que las ecuaciones que incluían tres pliegues cutáneos y dos perímetros explicaban más del 88% de la varianza del %GC, y las ecuaciones con menor número de medidas corporales explicaban menores varianzas ^14^. La Ecuación 3 es la más práctica pues sólo requiere tres medidas corporales y no incluye pliegues cutáneos. Si bien, esta ecuación con un SEE de 3.44 es clasificada como buena de acuerdo al Sistema de clasificación de Lohman ^19^, su rendimiento en el grupo de validación fue inferior a las ecuaciones 1 y 2, y explicó una menor varianza del %GC. La Ecuación 3 fue diseñada para la evaluación de grupos poblacionales en estudios epidemiológicos, donde usualmente es difícil contar con antropometristas estandarizados y mantener los equipos en óptimas condiciones.

Las ecuaciones antropométricas desarrolladas presentaron mejor rendimiento en la evaluación de la composición corporal que las ecuaciones foráneas. Las Ecuaciones 1, 2 y 3 produjeron resultados de composición corporal similares a los de hidrodensitometría y, presentaron una correlación y grado de acuerdo sustancial con el método de referencia, según los criterios de Landis ^20^. Por el contrario, las ecuaciones de Durning-Womersley, Jackson-Pollock y Ramírez-Torun mostraron diferencias significativas con el método de referencia, resultados similares a estudios previos ^9^^,^^14^^,^^15^^,^^21^^-^^23^. Los pobres resultados de las ecuaciones foráneas pueden obedecer a diferencias entre grupos étnicos en la densidad de los componentes corporales, en la distribución de la grasa corporal y/o en la proporcionalidad corporal, características que afectan el rendimiento de las ecuaciones ^9^^-^^11^. Así, las ecuaciones de Durning-Womersley y Jackson-Pollock desarrolladas en personas caucásicas, y las de Ramírez-Torun diseñadas en guatemaltecas, que son una mezcla de europeas y americanas nativas, mostraron ser inexactas en mujeres colombianas que se describen como una mezcla de europeas, africanas y americanas nativas ^24^^,^^25^.

De acuerdo al conocimiento de los autores, este estudio es único en desarrollar y validar ecuaciones antropométricas para estimar el %GC en mujeres Colombianas utilizando la hidrodensitometría como método de referencia. Otra fortaleza del estudio es el desarrollo de una ecuación antropométrica sin pliegues de grasa cutáneos, la cual se sugiere aplicar en grupos poblacionales en estudios epidemiológicos, donde los pliegues cutáneos generalmente no son medidos. El estudio tiene algunas limitaciones. El uso de una muestra a conveniencia, dado que la hidrodensitometría es una prueba que demanda a los sujetos estar bajo el agua 2 a 4 minutos respirando a través de una boquilla, por esto, la prueba no es práctica para evaluar muestras representativas de la población. Por lo tanto, se recomienda una interpretación cuidadosa de los resultados de las nuevas ecuaciones cuando se apliquen a la población general, en especial, cuando se utilicen con individuos que difieran significativamente de los sujetos de éste estudio, tanto en medidas corporales como en grupo étnico y nivel de actividad física. Otra limitación del estudio es el uso de un modelo de dos componentes que asume una densidad constante de la masa grasa y de la masa libre de grasa, se sabe que la densidad de estos componentes se modifica con la edad, la etnia y el nivel de actividad física ^17^. El uso de un modelo de cuatro componentes puede superar esta dificultad, pero no fue posible realizarlo en éste estudio por limitación de recursos tecnológicos. Es importante destacar que las Ecuaciones 1, 2 y 3 presentaron buen rendimiento en el grupo de validación, el cual tenía un IMC promedio de 23.7 kg/m^2^ y un %GC promedio de 31.3%. Se requieren estudios futuros para validar estas ecuaciones en grupos de mujeres con una mayor variación del IMC y del %GC.

En resumen, las ecuaciones desarrolladas, Ecuación 1 y 2, presentaron mejor rendimiento que las ecuaciones tradicionales para predecir la composición corporal en mujeres de Medellín-Colombia. La Ecuación 3 tiene más alto SEE y menor calidad estadística que las Ecuaciones 1 y 2, por lo que se sugiere aplicarla a grupos de personas en investigaciones epidemiológicas. Se recomienda una interpretación cuidadosa de los resultados de las nuevas ecuaciones cuando se aplique a individuos con características significativamente diferentes de los participantes de éste estudio
